# The Influence of L2 Proficiency on Bilinguals' Creativity: The Key Role of Adaptive Emotion Regulation Strategies During the COVID-19 Pandemic

**DOI:** 10.3389/fpsyg.2021.695014

**Published:** 2021-09-01

**Authors:** Yilong Yang, Shinian Wu, Jon Andoni Duñabeitia, Kexin Jiang, Yadan Li

**Affiliations:** ^1^Research Center for Linguistics and Applied Linguistics, Xi'an International Studies University, Xi'an, China; ^2^School of English Studies, Xi'an International Studies University, Xi'an, China; ^3^Department of English, Grand Valley State University, Allendale, MI, United States; ^4^Centro de Ciencia Cognitiva (C3), Antonio de Nebrija University, Madrid, Spain; ^5^AcqVA Aurora Center, The Arctic University of Norway, Tromsø, Norway; ^6^School of Telecommunications Engineering, Xidian University, Xi'an, China; ^7^MOE Key Laboratory of Modern Teaching Technology, Shaanxi Normal University, Xi'an, China; ^8^Shaanxi Normal University Branch, Collaborative Innovation Center of Assessment Toward Basic Education Quality at Beijing Normal University, Xi'an, China

**Keywords:** COVID-19, bilinguals, negative mood, emotion regulation, emotional creativity, cognitive creativity

## Abstract

The COVID-19 pandemic has brought severe impact on language learners' emotional states and their performance in creativity. Yet, their ability to regulate emotions is crucial for everyday functioning during times of crisis. The question of how adaptive emotion regulation (ER) strategies, which help an individual maintain appropriate and stable mood states, might affect bilinguals' creativity remains unexplored. The present study investigated this issue by measuring various indicators of the psychological impact of the COVID-19 pandemic, L2 proficiency, adaptive ER strategies, and bilinguals' cognitive creativity (CC) and emotional creativity (EC) during the pandemic. Results from a sample of 235 bilingual participants who completed a battery of survey instruments showed that: (1) bilinguals' negative mood significantly increased during the pandemic compared with their mood state before the pandemic; (2) their negative mood during the pandemic was positively associated with their adaptive ER strategies; (3) L2 proficiency had a direct effect on bilinguals' cognitive flexibility, CC, and EC; (4) L2 proficiency also indirectly influenced bilinguals' CC through cognitive flexibility. These results suggest that cognitive flexibility had a simple mediation effect on the association between L2 proficiency and CC. However, the current study further found that bilinguals had different cognitive patterns in EC. L2 proficiency influenced bilinguals' EC through cognitive flexibility indirectly only when adaptive ER strategies had a moderation effect on the association between cognitive flexibility and EC. However, this moderated mediation effect was not significant in CC. The current study implies that bilinguals' adaptive ER strategies played a distinct role in bilinguals' EC during the COVID-19 pandemic.

## Introduction

The COVID-19 pandemic has caused widespread panic and anxiety in the public (e.g., Banerjee, 2020; Jiloha, 2020; Nicomedes and Avila, 2020). Such a crisis might induce various behavioral, emotional, and physiological stress responses in people (Folkman, 2013). It would be interesting to explore the influence of bilingualism on creativity under such a circumstance with practical implications.

In recent decades, a positive effect of bilingualism on creativity has been confirmed by experimental research using various tasks that measure creative cognition (e.g., Lasagabaster, 2000; Simonton, 2008; Kharkhurin, 2010a,b; Kharkhurin, 2012; Leikin, 2013; Yang and Li, 2019; Yang et al., 2021). However, the domain of bilinguals' creativity in previous studies has mostly been limited to cognitive creativity (CC; Ma, 2009). To date, there has been no research investigating the influence of bilingualism on another major domain of creativity in bilinguals, i.e., emotional creativity (EC).

Previous studies have shown that EC is associated with the use of emotional regulation (ER) strategies. The proper use of ER strategies (i.e., adaptive ER strategies) helps individuals recover from adversities by interposing and adjusting their negative mood back to healthy levels (e.g., Aldao, 2013; Bonanno and Burton, 2013). The ability to regulate emotions is not only an ability of emotion management but also a cognitive ability. Therefore, the adaptive ER strategies might also play potential roles in bilinguals' creativity given the special context of the COVID-19 pandemic. However, the extent to which such strategies are effective in regulating both CC and EC during a major crisis remains unknown and warrants empirical research.

### Bilingualism, Cognitive Flexibility, and Cognitive Creativity

Creativity is defined as an individual's ability to generate both novel and useful ideas (e.g., Sternberg, 1999; Runco and Acar, 2012). It involves both executive (controlled) abilities and associative (spontaneous) abilities (e.g., Silvia et al., 2013; Beaty et al., 2014). Executive abilities entail top-down controls over thoughts through active switching between inhibition on mundane ideas and creative ideas; associative abilities, on the other hand, are related to spontaneous unfolding of creative ideas (Beaty et al., 2014). Second language (L2) learning seems to benefit both kinds of abilities. Bilinguals have more opportunities than monolinguals to practice and exercise cognitive control abilities, which help them manage two active language systems so as to solve misleading or conflicting language problems (Coderre et al., 2013). Multicultural experience, including learning an L2, is facilitative to one's creativity, especially in associative abilities (e.g., Lubart, 1999; Kharkhurin, 2008, 2010b; Leung et al., 2008; Simonton, 2008; Maddux and Galinsky, 2009; Leung and Chiu, 2010).

The role of bilingualism in creativity, mainly CC, has been already investigated in the literature. Previous studies have pointed to a general bilingual advantage in CC (e.g., Kharkhurin, 2012; van Dijk et al., 2019) as well as a positive effect of bilingualism on the various measures of CC in bilinguals, such as figurative creativity (e.g., Vaid et al., 2015), mathematical creativity (e.g., Lasagabaster, 2000; Simonton, 2008; Kharkhurin, 2010a), and language creativity (e.g., Kessler and Quinn, 1987; Ricciardelli, 1992b; Simonton, 2008; Leikin et al., 2014). Positive influence of bilingualism on CC has also been found in the three components of divergent thinking, i.e., originality (e.g., Konaka, 1997a; Kharkhurin, 2009), flexibility (e.g., Konaka, 1997a; Kharkhurin, 2009), and fluency (e.g., Ricciardelli, 1992a; Kharkhurin, 2008). Several factors of bilingualism that influence CC have been investigated, such as the age of L2 acquisition (e.g., Kharkhurin, 2008), cross-cultural experience (e.g., Simonton, 1997), and the length of exposure to L2 cultural settings (e.g., Kharkhurin, 2008). A positive role of L2 proficiency in bilinguals' CC has also been affirmed (e.g., Ricciardelli, 1992a; Simonton, 2008; Adesope et al., 2010; Leikin, 2013; Yang and Li, 2019; Yang et al., 2021). Language learners with different levels of L2 proficiency have been found to have different patterns of CC (Kharkhurin, 2011). More proficient bilinguals showed better performance in originality and greater violation of standard category properties than less proficient counterparts (Kharkhurin, 2011).

However, an important aspect of preceding studies to consider is that both languages of the bilinguals tested so far in the literature are mainly limited to Indo-European languages. Cognitive and linguistic processes in learning an L2 within the same language family could be quite different from those in learning a language of a different family (e.g., Bialystok and Miller, 1999). Studies have found that the native (L1) language of the bilinguals plays an important role in L2 learning, for example, showing different effects of age of acquisition and different levels of L2 attainment (e.g., Bialystok and Miller, 1999). Cognition might also be influenced by native language and culture (e.g., Levinson, 2003; Cook and Bassetti, 2011). Sabbagh et al. (2006), in constructing a theory of mind, for example, found that Asian bilingual children performed better on executive control in false-belief reasoning than their American counterparts. Therefore, the results of previous studies need to be strengthened through replication research that goes beyond Indo-European languages. To date, however, there are only a few studies exploring the influence of L2 proficiency on Chinese-English bilinguals' CC (see also Yang and Li, 2019; Yang et al., 2021). Chinese-English bilinguals have L1 in Sino-Tibetan languages and L2 in Indo-European languages.

Closely related to the effect of bilingualism on creativity is cognitive flexibility, one of the main components of cognitive control. It refers to an individual's ability to switch between different cognitive tasks (e.g., Miyake et al., 2000). Previous studies have confirmed the positive effect of bilingualism on cognitive flexibility measured by various tasks (Lehtonen et al., 2018). Some of these studies have suggested that individuals with higher degrees of bilingualism could switch between tasks by using a more efficient allocation of cognitive resources between tasks to select the correct responses than their counterparts with lower degrees of bilingualism (e.g., Abutalebi and Green, 2007; Luk et al., 2012; Hernández et al., 2013; De Baene et al., 2015). Direct evidence shows bilinguals with higher L2 proficiency levels outperform their counterparts with lower L2 proficiency in non-verbal cognitive flexibility (e.g., Bialystok et al., 2009; Wang and Cheng, 2016). In other words, existing research tends to affirm the overall positive role of L2 proficiency in bilinguals' cognitive flexibility, which in turn exerts a positive effect on CC.

One important ability in creativity is to have a broad attentional focus and switch between the alternatives and avoid relying on habitual thinking and fixed strategies in a task (e.g., Ashby et al., 1999). This ability is one of the three major indicators of creativity, known as flexibility. In a meta-analysis study, the results have shown that flexibility is associated with another two major indicators of creativity, i.e., fluency and originality (Nijstad et al., 2010). Evidence has suggested a cognitive flexibility pathway to creativity, which assumes that creative ideas and insight would be achieved through flexible switching among categories, sets, and approaches (e.g., Mednick, 1962; Amabile, 1983; Eysenck, 1993). Indeed, one important quality of creativity is the ability to break set or to overcome “functional fixedness” (e.g., Duncker, 1945; Wertheimer, 1945; Smith and Blankenship, 1991) and make new connections among distant ideas (e.g., Koestler, 1964; Simonton, 1999). Other studies have also linked cognitive flexibility with reduced levels of latent inhibition (e.g., Cohen et al., 2002; Carson et al., 2003; Dreisbach and Goschke, 2004). Reduced latent inhibition would lead to the possibilities of more distant associates and ideas in working memory, producing more original responses rather than habitual and dominant ones. Meanwhile, when people experience a change of emotions, they are self-aware and tend to interpret them in a conscious way. This kind of ability to interpret emotions is likely to increase cognitive flexibility in conflict resolution tasks (Averill and Nunley, 2010). The interpretation of emotions is also associated with another major domain of creativity, i.e., EC.

### Emotional Creativity and Emotion Regulation Strategies

EC is another major category of creativity. It is a battery of cognitive abilities and personality traits associated with originality and appropriateness of emotional experience (e.g., Averill, 1999; Ivcevic et al., 2007; Moltafet et al., 2018). The definition suggests that EC is related to both cognitive and emotional processes (Trnka et al., 2019). According to Averill (1999), EC has three components, namely, preparedness (i.e., understanding and learning from one's own and others' emotions), novelty (i.e., the ability to experience unusual emotions), and effectiveness/authenticity of experienced emotions (i.e., the skill to express emotions adroitly and honestly). EC plays a constructive role in self-regulation (Fuchs et al., 2007) because cognitive abilities inherent in EC help people understand their own emotions and exert adaptive control of these emotions. It has been reported that EC is interconnected with various cognitive abilities, such as self-understanding (Ivcevic et al., 2007), strategies for coping with stress (Averill, 1999), and cognitive creative abilities (Fuchs et al., 2007; Ivcevic et al., 2007). EC has been found to be a resource of the ability to react adaptively in situations of increased uncertainty and the ability to find new solutions under increased risk conditions (Frolova and Novoselova, 2015).

EC is yet still different from CC even though they are intimately associated with each other. Ma (2009) considers CC and EC as two major domains of creativity, suggesting that they are different psychological constructs. Empirical evidence based on statistical tests and confirmatory factor analyses has established a distinction between CC and EC (Trnka et al., 2016). Meanwhile, the literature also shows a positive relationship between EC and human creative performance because EC refers to people's ability to connect with the reasons and consequences of emotional responses at various stages of creative processes (e.g., Averill, 1999; Soroa et al., 2015). Therefore, CC and EC share some similarities. Evidence has suggested that higher EC is found to be associated with participants' increased involvement in creative leisure-time activities such as music composing, painting, and writing (e.g., Trnka et al., 2016; Kuška et al., 2020) and cognitive abilities involved in innovative performance such as idea generation and realization (Wang et al., 2015). More direct evidence has further found EC is interconnected with cognitive creative abilities (e.g., Fuchs et al., 2007; Ivcevic et al., 2007). EC may be associated with the Remote Associates Test (Ivcevic et al., 2007), a popular paradigm used to measure CC, and creativity styles (Fuchs et al., 2007). Since EC and CC are both influenced by emotions (Trnka et al., 2016), it is possible that bilinguals may share similar cognitive patterns in both domains of creativity. However, to the best of our ability, we did not find a study investigating the influence of bilingualism on EC.

ER strategies refer to behavioral and cognitive processes that adjust one's affective responses to internal and exogenous affect-eliciting events (e.g., Koole et al., 2015; Braunstein et al., 2017). ER strategies depend on an individual's ability to effectively modulate emotions using cognitive effort (e.g., Ochsner and Gross, 2005; Allard and Kensinger, 2014). ER strategies have thus been characterized as adaptive and maladaptive strategies (e.g., Aldao et al., 2010; Visted et al., 2018). Good psychological health is dependent on the use of adaptive ER strategies, which are flexible and situational abilities in managing one's emotions, whereas the rigid use of maladaptive ER strategies is related to a series of psychological problems such as depression (e.g., Aldao, 2013; Bonanno and Burton, 2013; Aldao et al., 2015). The reason behind this is obvious. When people experience negative mood, they would use adaptive ER strategies to manage their emotions so that negative mood would be interposed, readjusted, and bounced back to healthy levels. Studies have also demonstrated that older adults are generally more capable of regulating their emotions than younger adults (e.g., Blanchard-Fields et al., 2007; Blanchard-Fields, 2009). As people age, they show improvements in down-regulating feelings of disgust (e.g., Scheibe and Blanchard-Fields, 2009), in habitual use of problem-solving as an ER strategy (e.g., Vigouroux et al., 2017), and in using positive reappraisal (e.g., Shiota and Levenson, 2009; Lohani and Isaacowitz, 2014). Such an advantage is ascribed to the results of learning and practice, i.e., the accumulation of abilities in regulating various emotions over time (e.g., Blanchard-Fields et al., 2007; Blanchard-Fields, 2009). The richer or more diverse emotion contexts in which older adults live could foster their emotion interpretation processes, which in turn increases cognitive flexibility in the way they resolve conflicts (Averill and Nunley, 2010). Therefore, there can be a potential association between ER strategies and cognitive flexibility.

### The Current Study

**Figure 1:**
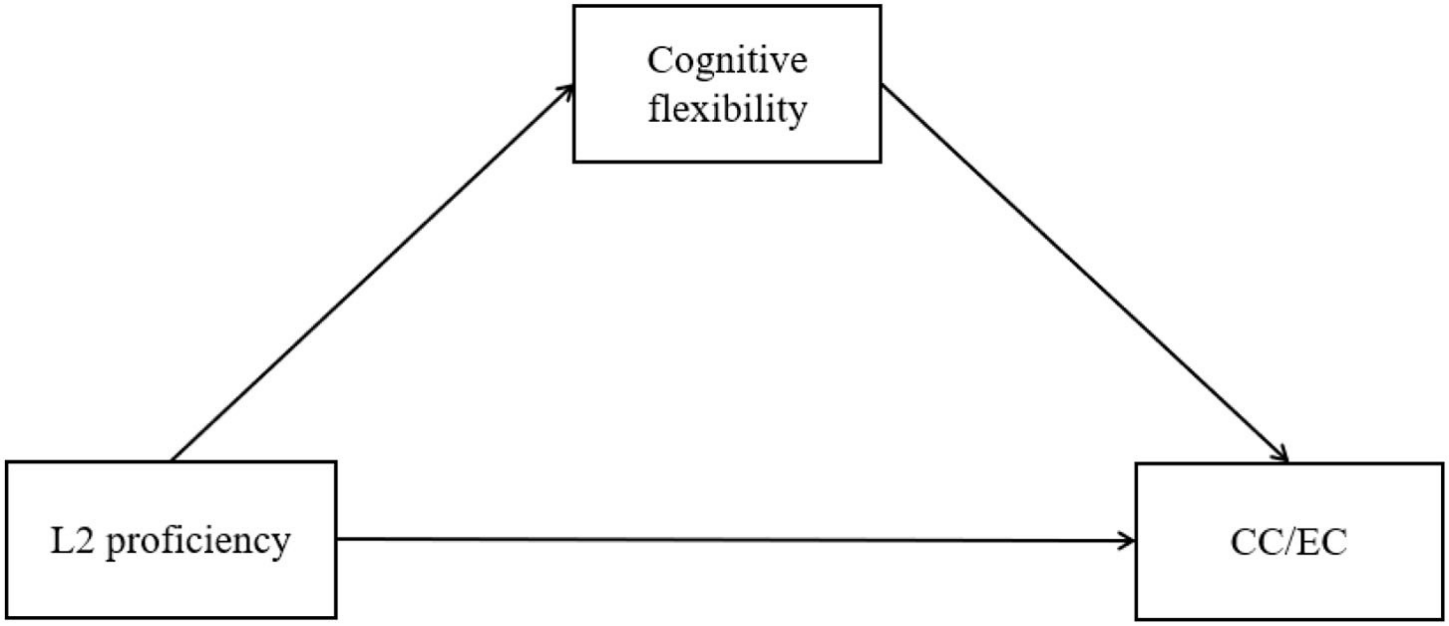
The simple mediation analysis using the PROCESS (Model 4) macro for SPSS.

As reviewed above, despite their similarities, CC and EC are essentially different psychological constructs. It is unknown whether L2 proficiency and ER strategies used during the COVID-19 pandemic would have the same influence on CC and EC. According to the dual pathway to creativity model, the flexibility pathway is one of two major pathways to achieve creativity (Nijstad et al., 2010). This theory posits that psychological and cognitive variables would influence creativity through their impact on cognitive flexibility (Nijstad et al., 2010). Following this line of research, the current study aimed to explore whether cognitive flexibility mediated the relationship between L2 proficiency and CC/EC in Chinese-English bilinguals. Moreover, the study also investigated whether bilinguals' use of adaptive ER strategies, which helps individuals maintain appropriate and stable mood states and hence psychological health, especially in times of crisis (e.g., the COVID-19 pandemic), exerted an influence on their CC and EC. Based on the literature and our goals, we hypothesized the following:

**H1**: Cognitive flexibility mediates the relationship between L2 proficiency and bilinguals' CC/EC (see model in Figure 1).

**Figure 2:**
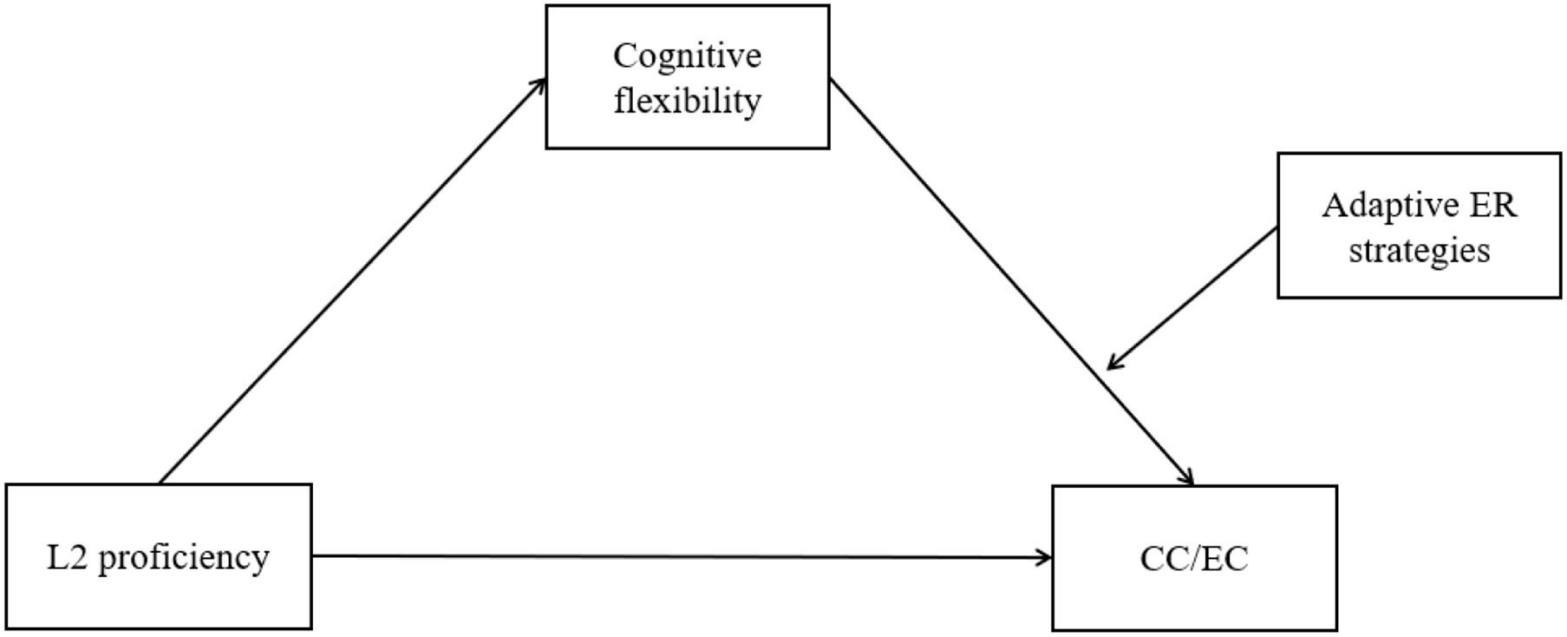
The moderated mediation analysis using the PROCESS (Model 14) macro for SPSS.

**H2**: Cognitive flexibility mediates the relationship between L2 proficiency and bilinguals' CC/EC when the indirect influence of L2 proficiency on CC/EC is moderated by adaptive ER strategies (see model in Figure 2).

## Materials and Methods

### Data Availability Statement

At the current stage, the original data are not made openly available in online repositories because of some restrictions in authors' institutions and/or confidentiality requirements of the programs where the authors work. Some of authors' institutional ethics committees require that the data be made available from them for researchers who meet the criteria for access to confidential data. Following the line of the 21-word solution (Simmons et al., 2012), we report how we determined our sample size, all data exclusions (if any), all manipulations, and all measures in the study.

### Participants

An increasing trend uses 20–80 research participants to test mediation (e.g., Koopman et al., 2015). This range of sample size is also advocated by Shrout and Bolger (2002). Studies show a sample size of 100 participants may be a justifiable minimum for moderation analysis (e.g., Maxwell, 2004; Koopman et al., 2015). In the current study, a total of 248 university students in China were recruited as study participants. All participants' first language (L1) was Mandarin Chinese and their second language (L2) was English. To ensure data validity, a subset of screening questions was used to determine whether the participants completed the investigation carefully, such as repeated selection (e.g., “please select the same choice in the previous question”), forced selection (e.g., “please select a certain item”), and reverse scoring (i.e., some items were reversely coded). We then screened the collected data to eliminate the results that failed to pass above validity check and abnormal answers (e.g., answers with regular patterns). The data of a total of 13 participants were discarded either because of incomplete answers or because they didn't pass the data screening. Therefore, the data of the remaining 235 participants (37 males, 198 females; mean age: 19.33 ± 0.65 years, range: 17.56–22.16 years) were used in the analysis. According to a background survey on L2 learning, the participants had a mean age of L2 acquisition of 8.66 (± 2.08) years (range: 3–13 years), and were spending an average of 1.49 (± 1.42) hours in learning L2 per day, and using L2 for 0.94 (± 1.06) hours a day. Based on a 6-point Likert scale, the participants reported a confidence level of 3.28 (± 1.02) for L2 and a positive attitude of 3.76 (± 1.31) toward L2 courses. According to a demographic survey, participants were all right-handed with no known neurological and psychiatric disorders or substance abuse. All participants signed a written informed consent form and were paid once the study was finished. The study was approved by the Academic Committee of the Ministry of Education Key Laboratory of Modern Teaching Technology, Shaanxi Normal University in China.

### Measures

#### LexTALE Vocabulary Test

Participants' L2 proficiency was measured using an English vocabulary test, i.e., LexTALE (Lemhöfer and Broersma, 2012). The LexTALE vocabulary test is used as a valid and reliable predictor of English vocabulary and English language proficiency (Lemhöfer and Broersma, 2012). Test instructions were shown to the participants before the test administration. During the test, participants would see a string of letters in each trial and were asked to decide whether the string was an existing British English word by clicking “yes” or “no.” There were 60 trials (plus 3 additional practice trials) for the whole vocabulary test. Following the test instructions, the trials would be presented in a fixed sequence. There was no time limit for each trial but the participants were not allowed to use any outside materials or information during the test. A question prior to the test showed none of the participants had taken LexTALE before.

#### The Cognitive Flexibility Inventory

The Cognitive Flexibility Inventory (CFI; Dennis and Vander Wal, 2010) was used to measure the participants' cognitive flexibility. The CFI was developed to measure the type of cognitive flexibility necessary for individuals to challenge and replace maladaptive thoughts with more balanced and adaptive thinking. It measures three facets of cognitive flexibility: (a) the ability to generate multiple alternative solutions to difficult situations; (b) the ability to perceive multiple alternative explanations for life occurrences and human behavior; and (c) the tendency to perceive difficult situations as controllable.

The CFI consists of 20 items on a 5-point Likert scale (1 = “never” to 5 = “very often”). The sum of the 20 items is the participants' score of performance in cognitive flexibility. The validity and reliability of the CFI measuring cognitive flexibility have been well established (Dennis and Vander Wal, 2010). Its Chinese version has also been tested and has satisfying validity and reliability (Wang et al., 2016). The internal consistency reliability of the CFI in the current study was satisfactory (Cronbach's α = 0.83).

#### The Runco Ideational Behavior Scale

The Runco Ideational Behavior Scale (RIBS; Runco and Acar, 2012) was used to measure the participants' performance in CC. The RIBS was developed based on the belief that ideas could be regarded as the products of original, divergent, and creative thinking. It can thus be used as a measure of creative ideation. Most items of the RIBS describe actual behaviors (i.e., overt activities and actions) that reflect one's use of, appreciation of, and skill with ideas. The scale has been tested and appears to be a reliable instrument for use with individuals and groups. The RIBS consists of 23 items on a 5-point Likert scale (1 = “never” to 5 = “very often”). The sum of the 23 items is the participants' score of performance in CC. The internal consistency reliability of the RIBS in the current study was satisfactory (Cronbach's α = 0.85).

#### The Emotional Creativity Inventory

The EC Inventory (ECI; Averill, 1999) was used to measure the participants' performance in EC. The ECI measures three facets of EC, namely, emotional preparedness (i.e., understanding and learning from one's own and others' emotions), novelty of emotional experiences (i.e., the ability to experience unusual emotions), and effectiveness/authenticity of experienced emotions (i.e., the skill to express emotions adroitly and honestly). The ECI is widely used and has cross-cultural adaptability. The Chinese version of the ECI has also shown good validity and reliability (Wang and Yan, 2017) and was adopted for the present study. The Chinese version of the ECI consists of a total of 26 items and is further divided into three subscales, namely, emotional preparedness, novelty of emotional experiences, and effectiveness/authenticity of experienced emotions. The Chinese version of the ECI also uses a 5-point Likert scale (1 = “strongly disagree” to 5 = “strongly agree”). The internal consistency reliability of the ECI in the current study was satisfactory (Cronbach's α = 0.81).

#### The Positive and Negative Affect Schedule

The Negative Affect subscale of the Positive and Negative Affect Schedule (PANAS-negative; Watson et al., 1988) was used to measure the participants' negative mood. The PANAS-negative contains a total of 10 items measured on a 5-point Likert scale (1 = “very slightly” to 5 = “extremely”). The higher the scores on the PANAS-negative, the higher the negative mood or affect. The internal consistency reliability of the PANAS-negative in the current study was satisfactory (Cronbach's α = 0.86).

#### The Impact of Event Scale-Revised

The Impact of Event Scale (IES; Horowitz et al., 1979) was used to measure the psychological impact of a particular life event on the participants. The IES was revised and included a hyper-alert component (IES-R; see Christianson and Marren, 2012 for a review; Weiss and Marmar, 1997). The IES-R consists of a total of 22 items divided into three subscales: avoidance symptoms, invasive symptoms, and high arousal symptoms. The IES-R uses a 5-point Likert scale (0 = “not at all” to 4 = “extremely”). The validity and reliability of IES-R measuring the extent of psychological impact of public health crises have been well-validated in the Chinese population (Zhang, 2014; Wang et al., 2020). The specific life event of IES-R in the current study was the COVID-19 pandemic. The internal consistency reliability of the IES-R in the current study was satisfactory (Cronbach's α = 0.89).

#### The Depression Anxiety Stress Scale 21

The Depression Anxiety Stress Scale (DASS) was used to measure participants' depression, anxiety, and stress (Lovibond and Lovibond, 1995). The current study used the simplified version of DASS (i.e., DASS-21; Antony et al., 1998; Crawford and Henry, 2003). DASS-21 consists of a total of 21 items divided into three subscales: depression, anxiety, and stress. Measured on a 4-point Likert scale (0 = “not at all” to 3 = “nearly every day,” it has the same factor structure with the same reliability and validity as the full version (i.e., DASS). The internal consistency reliability of DASS-21 used in the current study was satisfactory (Cronbach's α = 0.90).

#### The Cognitive Emotion Regulation Questionnaire

The Cognitive Emotion Regulation Questionnaire (CERQ) was used to measure participants' emotion regulation strategies (Garnefski et al., 2001). CERQ consists of a total of 36 items divided into nine subscales, each of which represents a cognitive emotion regulation strategy that people would likely use after experiencing negative life events or situations. The nine subscales are self-blame, acceptance, rumination, putting into perspective, positive refocus, refocus on planning, positive reappraisal, catastrophizing, and blaming others. Each subscale has four items and uses a 5-point Likert scale (1 = “almost never” to 5 = “almost always”). The nine emotion regulation strategies subscales could be classified into two more general types of strategies, i.e., adaptive cognitive emotion regulation strategies (i.e., CERQ-adaptive) and maladaptive cognitive emotion regulation strategies (i.e., CERQ-maladaptive). The Chinese version of CERQ has been well validated in the Chinese population (Zhu et al., 2007). The current study focused on the participants' use of CERQ-adaptive. The CERQ-adaptive consists of four subscales, namely putting into perspective, positive refocus, refocus on planning, and positive reappraisal. Higher scores on these four subscales represent a greater frequency of using adaptive cognitive emotion regulation strategies. The internal consistency reliability of the CERQ-adaptive in the current study was satisfactory (Cronbach's α = 0.80).

### Procedure and Statistical Analysis

The psychological measures of the participants in the current study were distributed both before and during the COVID-19 pandemic. Before the outbreak of the COVID-19 pandemic (October 2019), the participants completed a demographic survey and a negative mood test (PANAS). During the lockdown in the height of the pandemic (July 2020), the participants completed an L2 proficiency test (LexTALE), a cognitive flexibility test (CFI), a cognitive creativity test (RIBS), an emotional creativity test (ECI), a negative mood re-test (PANAS), a psychological impact test (IES-R), a test on depression, anxiety and stress (DASS-21), and a test on adaptive ER strategies (CERQ) on their computers or other electronic devices via an online survey platform (http://www.wjx.cn), a well-known and widely used research service.

The descriptive statistics and correlation analyses were performed using IBM Statistical Package for Social Sciences (SPSS) version 25.0 (SPSS Inc., Chicago, USA). The PROCESS (v. 3.5) macro for SPSS was used to test the mediation effect and moderated mediation effect (Preacher and Hayes, 2004; Hayes, 2013). Two steps were taken to test the two hypotheses raised for this study. First, we conducted the simple mediation analysis for CC and EC, respectively. Then we conducted the moderated mediation analysis for CC and EC, respectively.

#### Test of Mediation

Hypothesis 1 suggested that L2 proficiency positively influenced bilinguals' CC/EC through cognitive flexibility. We tested this simple mediation hypothesis (Figure 1) using the PROCESS (v. 3.5; Model 4) macro for SPSS, with a bootstrapping sample size of 5,000 and 95% confidence intervals (CIs). We set L2 proficiency as the independent variable, cognitive flexibility as the mediation variable, and CC/EC as the dependent variable. The simple mediation analysis allows us to reveal the direct association between L2 proficiency and bilinguals' CC/EC, in the form of regression weights, and calculate the indirect path, that is, the influence of L2 proficiency on bilinguals' CC/EC through cognitive flexibility.

#### Test of Moderated Mediation

Hypothesis 2 suggested that, while L2 proficiency enhanced bilinguals' CC/EC through cognitive flexibility, bilinguals' adaptive ER strategies would play a moderation role in the relationship between cognitive flexibility and CC/EC. We tested this moderated mediation hypothesis (Figure 2) using the PROCESS (v. 3.5; Model 14) macro for SPSS, with a bootstrapping sample size of 5,000 and a 95% confidence level. We set L2 proficiency as the independent variable, cognitive flexibility as the mediation variable, adaptive ER strategies as the moderation variable, and CC/EC as the dependent variable. The moderated mediation analysis allows us to assess whether an indirect effect in a mediation model is moderated and demonstrate whether the mediation effect exists at certain levels of the moderator.

## Results

### Descriptive Statistics and Correlation Analysis

**Figure 3:**
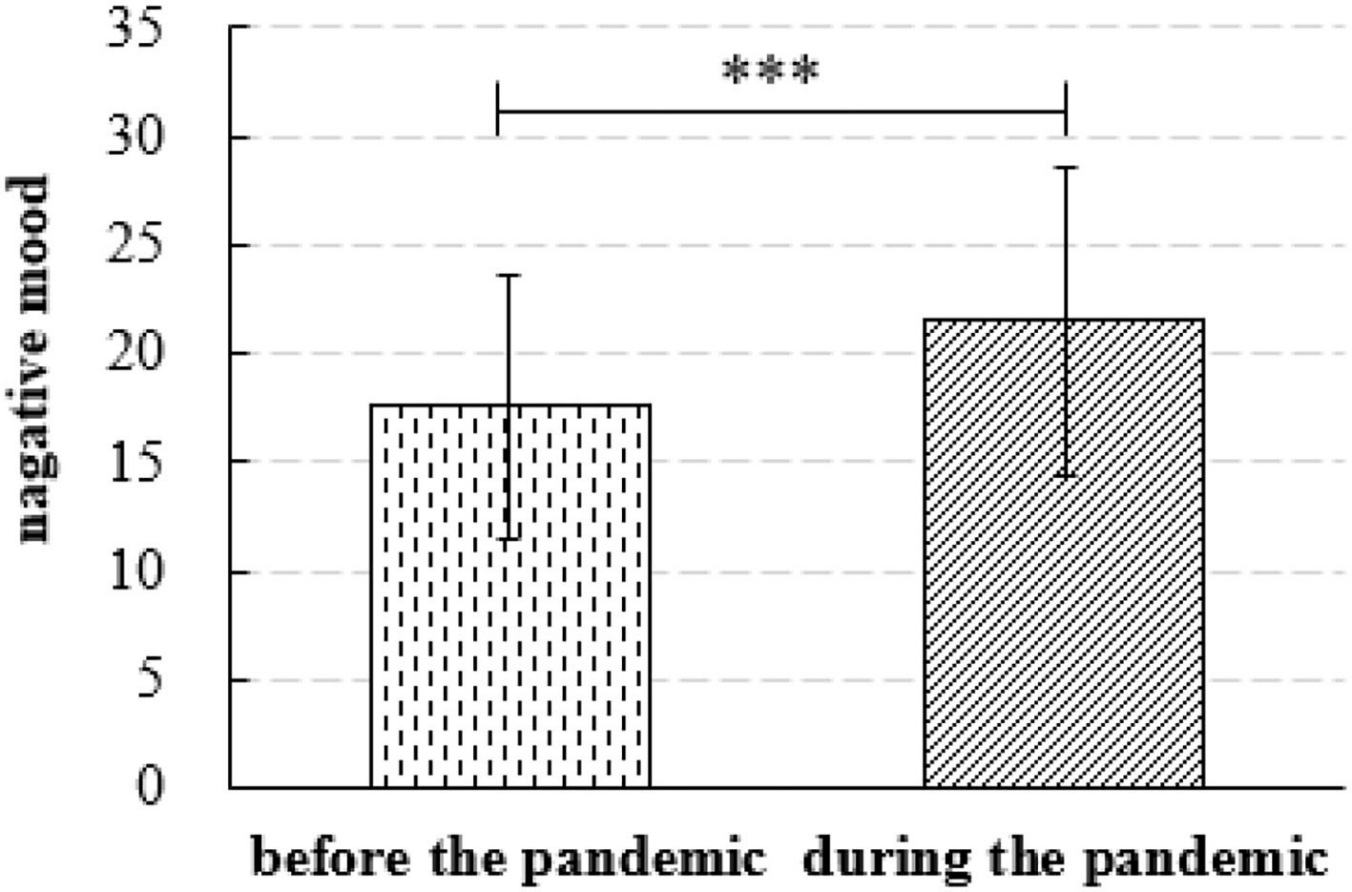
Mean scores of participants' negative mood measured by the PANAS-negative. Error bars represent standard deviation (*SD*). ***p < 0.001.

Measures on the participants' mood states include PANAS-negative, IES-R (42.83 ± 14.04), depression (DASS; 10.83 ± 3.67), anxiety (DASS; 9.79 ± 3.08), and stress (DASS; 9.84 ± 3.17). The paired *t*-test showed a significant increase (*p* < 0.001) of PANAS-negative scores for the participants during the COVID-19 pandemic (21.50 ± 7.15) than before the pandemic (17.58 ± 6.04; Figure 3). During the pandemic, scores of PANAS-negative positively were in positive associations with the scores of IES-R (*r* = 0.38, *p* < 0.001), depression (*r* = 0.62, *p* < 0.001), anxiety (*r* = 0.57, *p* < 0.001), and stress (*r* = 0.56, *p* < 0.001). During the pandemic, scores of adaptive ER strategies were in positive associations with scores of PANAS-negative (*r* = 0.16, *p* < 0.05), IES-R (*r* = 0.14, *p* < 0.05), depression (*r* = 0.15, *p* < 0.05), and anxiety (*r* = 0.17, *p* < 0.01).

Table 1 shows the means, standard deviations, and correlations of the main variables tested during the pandemic in the present study. L2 proficiency was in positive associations with cognitive flexibility (*r* = 0.14, *p* < 0.05), CC (*r* = 0.26, *p* < 0.001), EC (*r* = 0.31, *p* < 0.001), and adaptive ER strategies (*r* = 0.17, *p* < 0.05). Cognitive flexibility was in positive associations with CC (*r* = 0.36, *p* < 0.001), EC (*r* = 0.18, *p* < 0.01), and adaptive ER strategies (*r* = 0.31, *p* < 0.001).

**Table 1:** Means, standard deviations, and correlations between variables.

**Variables**	***M* ±*SD***	**1**	**2**	**3**	**4**	**5**
**1**. L2 proficiency	34.62 ± 4.50	1				
**2**. Cognitive flexibility	67.99 ± 8.20	0.14^*^	1			
**3**. EC	87.54 ± 9.21	0.31^***^	0.18^**^	1		
**4**. CC	74.32 ± 9.58	0.26^***^	0.36^***^	0.52^***^	1	
**5**. Adaptive ER strategies	53.11 ± 5.84	0.17^*^	0.31^***^	0.17^*^	0.21^**^	1

*EC, emotional creativity; CC, cognitive creativity; ER, emotion regulation*.

* *p < 0.05*,

** *p < 0.01*,

*** *p < 0.001*.

### Mediation and Moderation Analysis

#### Cognitive creativity

**Figure 4:**
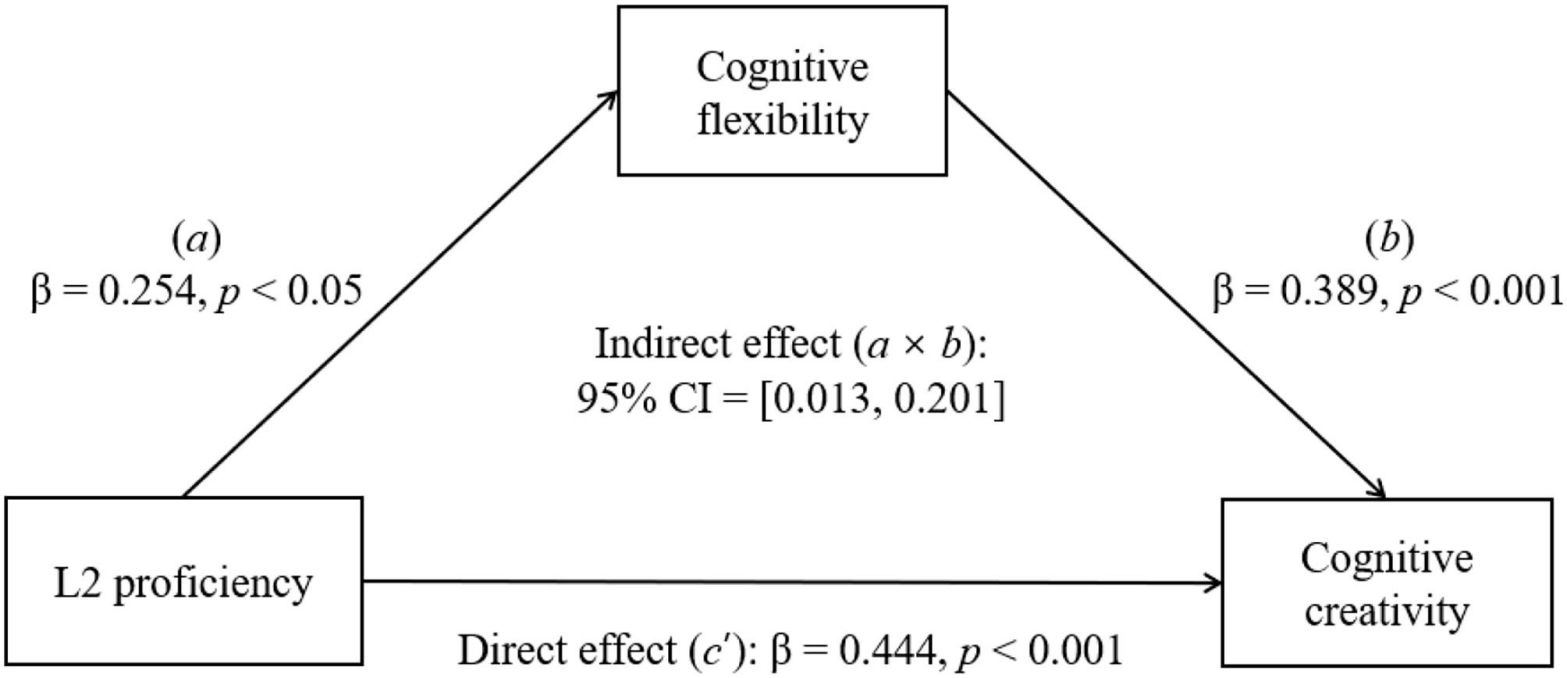
Mediating role of cognitive flexibility in the association between L2 proficiency and CC (Model 4). The depicted is the path diagram of the simple mediation analysis showing the influence of L2 proficiency on the bilinguals' CC through cognitive flexibility. Statistical results were satisfied for a mediation effect: Path *a*, Path *b*, Path *c'*, and Path *a* × *b* (β = 0.099, *SE* = 0.048, 95% CI = [0.013, 0.201]) were all significant.

We firstly used simple mediation analysis (Model 4) to test the role of cognitive flexibility (see the hypothetical model in Figure 1). The model was significant and accounted for a significant proportion of the variance in bilinguals' CC [*R*^2^ = 0.174, *F*(2, 233) = 24.380, *p* < 0.001]. L2 proficiency was in positive associations with cognitive flexibility (β = 0.254, *SE* = 0.118, *p* < 0.05) and CC (β = 0.444, *SE* = 0.128, *p* < 0.001). Meanwhile, cognitive flexibility was in positive association with CC (β = 0.389, *SE* = 0.070, *p* < 0.001). Moreover, L2 proficiency significantly influenced bilinguals' CC through cognitive flexibility (β = 0.099, *SE* = 0.048, 95% CI = [0.013, 0.201]). These results are shown in Figure 4. We then incorporated adaptive ER strategies into this model as a moderation variable to investigate their influence. A moderated mediation analysis was performed using Model 14 (see the hypothetical model in Figure 2), in which adaptive ER strategies was a moderator in the association between cognitive flexibility and CC. However, the index of moderated mediations was not significant (β = −0.007, *SE* = 0.004, 95% CI = [−0.016, 0.0001]). This suggests that bilinguals' adaptive ER strategies were not playing a moderation role in the association between cognitive flexibility and CC.

#### Emotional Creativity

Following the analysis of CC, initially we used Model 4 to test the simple mediation effect in EC (Figure 1). The results showed that L2 proficiency was in positive associations with cognitive flexibility (β = 0.254, *SE* = 0.118, *p* < 0.05) and EC (β = 0.591, *SE* = 0.128, *p* < 0.001). Cognitive flexibility was correlated with EC (β = 0.153, *SE* = 0.070, *p* < 0.05). Though the direct effect was significant, the indirect effect (i.e., the influence of L2 proficiency on EC through cognitive flexibility) was nonsignificant (β = 0.039, *SE* = 0.028, 95% CI = [−0.001, 0.104]). This result suggested that the simple mediation mechanism in bilinguals' EC was non-significant.

**Figure 5:**
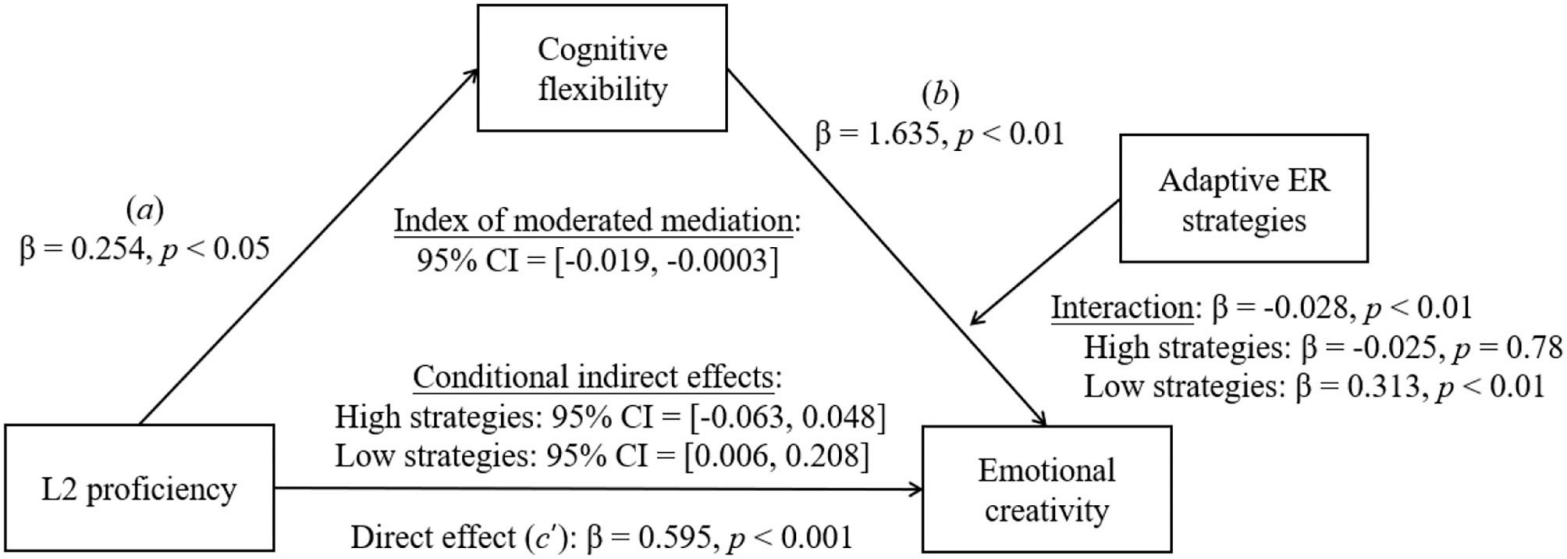
Moderated mediation roles of cognitive flexibility and adaptive ER strategies in bilinguals' EC. The depicted is the path diagram of moderated mediation analysis showing both the direct and the indirect effect of L2 proficiency on bilinguals' EC. Statistical results were satisfied for a moderated mediation effect: Path *a*, Path *b*, and Path *c'* were all significant. The adaptive ER strategies had a moderation effect on Path *b* when their use was at a low level (−1 *SD*).

**Figure 6:**
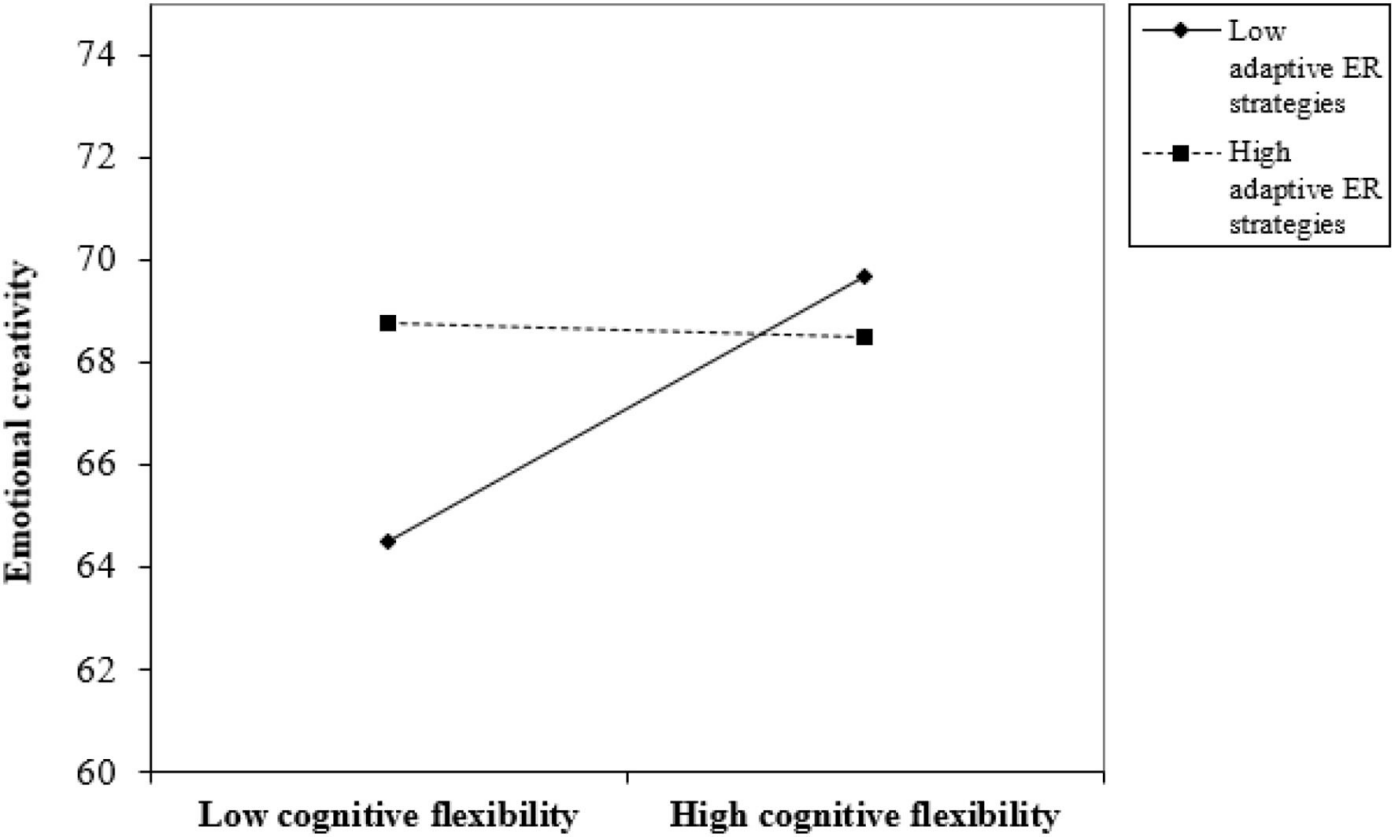
Interaction plot of the moderating effect of adaptive ER strategies on the relationship between cognitive flexibility and EC.

We then proceeded to conduct moderated mediation analysis using Model 14 to test the mediation role of cognitive flexibility and moderation role of adaptive ER strategies (see the hypothetical model in Figure 2). The model was significant and accounted for a significant proportion of the variance in bilinguals' EC [*R*^2^ = 0.150, *F*(2, 233) = 10.108, *p* < 0.001; Figure 5]. L2 proficiency was positively associated with cognitive flexibility (β = 0.254, *SE* = 0.118, *p* < 0.05) and EC (β = 0.595, *SE* = 0.127, *p* < 0.001). Meanwhile, cognitive flexibility was positively associated with EC (β = 1.635, *SE* = 0.533, *p* < 0.01). However, this association was moderated by bilinguals' adaptive ER strategies (β = −0.028, *SE* = 0.010, *p* < 0.01; Figure 6). The moderation effect of adaptive ER strategies was significant when they were at low (−1 *SD*; β = 0.313, *SE* = 0.098, *p* < 0.01) and medium (β = 0.144, *SE* = 0.073, *p* < 0.05) levels. L2 proficiency significantly influenced bilinguals' EC through cognitive flexibility only when adaptive ER strategies were at a low level (−1 *SD*; β = 0.079, *SE* = 0.052, 95% CI = [0.006, 0.208]). The index of moderated mediation was significant (β = −0.007, *SE* = 0.005, 95% CI = [−0.019, −0.0003]). These results suggested a multifaceted construct of EC based on different cognitive mechanisms.

## Discussion

Previous studies have shown that bilinguals had better performance in creativity than monolinguals (e.g., Lasagabaster, 2000; Simonton, 2008; Kharkhurin, 2010a,b; Kharkhurin, 2012; Leikin, 2013) and language proficiency has played a positive role (e.g., Ricciardelli, 1992a,b; Simonton, 2008; Adesope et al., 2010; Leikin, 2013). In the current study, we further investigated the potential influence of cognitive flexibility and adaptive ER strategies on bilinguals' CC and EC during the COVID-19 pandemic based on simple mediation and moderated mediation analyses. Our study showed that there was significantly increased negative mood in bilinguals during the pandemic compared with the period before the pandemic, as expected given the socioeconomic, psychological and health impact from the pandemic. Bilinguals' negative mood during the pandemic was associated with their adaptive ER strategies. We also found that L2 proficiency influenced bilinguals' CC both directly and indirectly through cognitive flexibility. Moreover, L2 proficiency influenced bilinguals' EC both directly and indirectly through cognitive flexibility only when their adaptive ER strategies were affecting the indirect influence of L2 proficiency on EC. However, we didn't find a significant role of bilinguals' adaptive ER strategies in their CC. This suggests that adaptive ER strategies played a key role in bilinguals' EC and made a different impact on and even led to different cognitive mechanisms underlying CC and EC.

### The Psychological Influence of the COVID-19 Pandemic on Bilinguals

Our study found that bilinguals showed significantly increased negative mood during the COVID-19 pandemic than before the pandemic. This finding is consistent with the results of recent studies on the psychological states of the public during the COVID-19 epidemic (e.g., Cao et al., 2020). We found that participants' negative mood (measured by PANAS-negative) was in positive association with psychological impact (measured by IES-R) during the pandemic. This result is in line with previous studies (e.g., Morina and von Collani, 2006). The current study also found that bilinguals with higher levels of negative mood (measured by PANAS-negative) during the pandemic showed higher levels of depression, anxiety, and stress. Such a result is consistent with other studies on the validity and measurement properties of PANAS based on large samples (e.g., Crawford and Henry, 2004; Oei et al., 2013). Our results provide a more comprehensive and more specific description of negative mood in bilinguals during the COVID-19 epidemic.

The purpose of CERQ is to assess cognitive ER strategies people use when they are experiencing negative life events (e.g., Garnefski et al., 2001). We found that bilinguals' negative mood was positively associated with their adaptive ER strategies. Previous studies have suggested that participants' ratings in depression and anxiety significantly correlate with all nine subscales of CERQ (e.g., Duan and Zhu, 2020). Other studies have also shown that ratings of several subscales of adaptive ER strategies are also correlated with depression (e.g., Jermann et al., 2006; McKinnon et al., 2020). When people have negative mood, they would use adaptive ER strategies to manage their emotions. In doing so, negative mood would be interposed, readjusted, and bounce back to healthy levels. Therefore, the use of cognitive ER strategies is significantly correlated with psychological well-being in various population and age groups (e.g., Garnefski et al., 2002, 2009b,c; Garnefski et al., 2009a).

### The Mediation and Moderation Mechanisms in Bilinguals' CC and EC

Although previous studies have suggested that L2 proficiency is associated with cognitive flexibility (e.g., Sun et al., 2019) and bilinguals' CC (e.g., Ricciardelli, 1992a,b; Simonton, 2008; Adesope et al., 2010; Leikin, 2013; Yang and Li, 2019; Yang et al., 2021), no research has explored the potential relationships among L2 proficiency, cognitive flexibility, and bilinguals' CC. Here we extended previous research on bilinguals' creativity by incorporating the influence of cognitive flexibility, which has been found to positively correlate with CC. In addition, we found that EC was related to several cognitive variables (i.e., L2 proficiency and cognitive flexibility) as CC was. Evidence has suggested that CC and EC are related to both cognitive and emotional variables and share some similarities (e.g., Trnka et al., 2016; Kuška et al., 2020) since they are partly overlapping psychological constructs that involve similar skills and attitudes (e.g., Fuchs et al., 2007; Trnka et al., 2016). Importantly, both CC and EC may be associated with creative behavior. Therefore, we expected that EC might display similar cognitive patterns as CC does.

First, our study showed that L2 proficiency had a direct influence on bilinguals' CC and EC. Existing evidence points to a bilingual advantage in language learners' CC (e.g., Ricciardelli, 1992a; Simonton, 2008; Adesope et al., 2010; Leikin, 2013; Yang and Li, 2019; Yang et al., 2021). Comparative studies on monolinguals, unbalanced bilinguals, and balanced bilinguals suggest a positive correlation between bilingualism and bilingual speakers' cognitive creative thinking (e.g., Konaka, 1997b; Simonton, 2008; Leikin, 2013; Sampedro and Peña, 2019). Some studies have further confirmed the positive role of L2 proficiency in CC (e.g., Ricciardelli, 1992a,b; Simonton, 2008; Adesope et al., 2010; Leikin, 2013). The current study extended this line of research by expanding the experimental sample to bilinguals whose L1 is in Sino-Tibetan languages and L2 in Indo-European languages (see also Lee and Kim, 2011; Yang and Li, 2019; Yang et al., 2021), and confirmed a positive correlation between L2 proficiency and bilinguals' performance of CC.

The current study also extended previous research by finding that L2 proficiency had a direct effect on bilinguals' EC. Although no such direct evidence has been reported in the research literature, indirect evidence could support this result. EC is a battery of cognitive abilities and personality traits that are associated with originality and appropriateness of emotional experience (e.g., Averill, 1999; Ivcevic et al., 2007; Moltafet et al., 2018). Previous studies have shown that CC and EC share some similarities (e.g., Averill, 1999; Fuchs et al., 2007; Ivcevic et al., 2007; Soroa et al., 2015; Wang et al., 2015; Trnka et al., 2016; Kuška et al., 2020). Therefore, we had expected that L2 proficiency would have similar influence on EC.

Second, the current study extended previous research by showing that L2 proficiency had an indirect influence on bilinguals' CC through cognitive flexibility. We tested the indirect influence of L2 proficiency on CC using simple mediation analysis. Statistical results fit well with the flexibility pathway to creativity proposed by previous studies (De Dreu et al., 2008; Nijstad et al., 2010). The findings suggest that proficient bilinguals have better performance in cognitive flexibility than monolinguals (Bialystok et al., 2009, 2012). A possible reason is that bilinguals need to switch between two languages and inhibit activated non-target language when they are processing languages (Dong and Xie, 2014). Bilinguals use a language control system, which is part of the general cognitive control, to inhibit the activated non-target language in comprehending and producing target language (e.g., Dong and Xie, 2014; Borragan et al., 2018). Such continuous exercise of inhibition in language processing strengthens the general cognitive control, in which cognitive flexibility is a key component. Proficient L2 learners gain more experience with language and cultural switching or translation. Therefore, their cognitive flexibility is more frequently trained and better improved as a result.

Another explanation of this finding lies in the mechanism of creativity. In the domain of creative cognition, one popular theory explaining the cognitive mechanism of creativity is the dual pathway model, which assumes that psychological states or traits influence CC through their impact on flexibility as one of the two major cognitive pathways (De Dreu et al., 2008; Nijstad et al., 2010). The flexibility pathway to creativity represents the possibility of problem-solving and achieving creative ideas and insights through flexible switching among approaches, categories, and sets (e.g., Mednick, 1962; Amabile, 1983; Eysenck, 1993). Previous research has shown that CC is often correlated with “breaking set,” overcoming “functional fixedness” (e.g., Duncker, 1945; Wertheimer, 1945; Smith and Blankenship, 1991), and making connections among remote ideas (e.g., Koestler, 1964; Simonton, 1999). In creative cognition, it is important for people to have a broad attentional focus and be able to switch between different alternatives to the task instead of relying on habitual thinking and fixed strategies (e.g., Ashby et al., 1999). Biological evidence suggested that dopamine releases would cause an increase in cognitive flexibility, which in turn contributed to CC (Nijstad et al., 2010). However, no existing research has explored the potential role of L2 proficiency as the independent variable in the dual pathway to creativity model. Here, we extended this line of research.

Third, the current study showed that bilinguals' L2 proficiency had a positive influence on their EC through cognitive flexibility only when their adaptive ER strategies were playing a moderation role in the indirect influence (i.e., L2 proficiency → cognitive flexibility → EC). There are several associations that could explain this moderated mediation mechanism of cognition in EC. On the one hand, cognitive flexibility was associated with bilinguals' adaptive ER strategies. When people experience the change of emotions, they are self-aware and they are interpreters of their own emotional experiences. This process would increase flexibility in the way people resolve conflicts (Averill and Nunley, 2010). Since ER strategies are related to both emotional and cognitive variables, the finding of a significant positive association between cognitive flexibility and bilinguals' ER was expected.

On the other hand, bilinguals' adaptive ER strategies were associated with their EC. This finding is consistent with direct and indirect evidence in previous studies. Since EC includes cognitive abilities that are important for understanding people's own emotions and the adaptive control of emotions (Trnka et al., 2019), EC was found to be important in behavioral self-regulation (Fuchs et al., 2007). Studies that focused on older adults' cognitive processing and control of emotions have shown that they have improved performance in various subscales of adaptive ER strategies than younger adults (e.g., John and Gross, 2004; Fuchs et al., 2007; Blanchard-Fields, 2009; Shiota and Levenson, 2009; Lohani and Isaacowitz, 2014; Masumoto et al., 2016; Vigouroux et al., 2017). This advantage is due to the accumulating experience of regulating various emotions in life. Therefore, older adults, compared with younger ones, are considered to have more complex, flexible, and emotionally mature functioning (Blanchard-Fields et al., 2007). Previous studies also have suggested that bilinguals have a richer and more diverse range of emotions compared with monolinguals (e.g., Alqarni and Dewaele, 2020). In the course of regulating rich and diverse emotions in the two languages, bilinguals might also accumulate the experience of using ER strategies in the process of L2 learning in a similar way as older adults. Since both ER and EC are associated with emotional and cognitive variables, it is reasonable to suggest that they are positively correlated with each other.

### The Different Effects of Adaptive ER Strategies on CC and EC

While the current study suggested that L2 proficiency had a positive influence on bilinguals' CC and EC, it further showed that bilinguals' adaptive ER strategies had played distinct roles in their CC and EC.

There might be a potential connection between CC and EC. The current study found that both the bilinguals' CC and EC were associated with their L2 proficiency, cognitive flexibility, and adaptive ER strategies. Though EC is essentially related to emotion, various cognitive abilities are also required to regulate emotions. One of these important abilities is cognitive flexibility. Therefore, it came as no surprise that a significant connection between EC and cognitive flexibility was observed in the current study. Also, cognitive flexibility plays a critical role in a wide range of cognitive and emotional processes, including language processing, CC, and ER. In the current study, L2 proficiency was found to influence bilinguals' CC through cognitive flexibility (see Figure 4). L2 proficiency also influenced bilinguals' EC through cognitive flexibility when ER strategies were moderating the direction influence of L2 proficiency on EC (see Figure 5). In these relationships, cognitive flexibility is the key variable connecting CC, EC, and adaptive ER strategies. Moreover, previous studies have explored the relationship between CC and EC. They have suggested a series of connections between them. For example, the novelty subscale of ECI has been found to be associated with the Remote Associates Test (e.g., Ivcevic et al., 2007). Furthermore, preceding research has shown that EC predicts the individual's ability to find new solutions under increased risk conditions and to respond adaptively in cases of increased uncertainty (e.g., Frolova and Novoselova, 2015). EC is found to be correlated with creativity styles in college students (e.g., Fuchs et al., 2007).

However, as pointed out previously, CC and EC are apparently different psychological constructs. The current study found that CC and EC had different associations with other psychological variables. While both the bilinguals' CC and EC were related to L2 proficiency, cognitive flexibility, and adaptive ER strategies, their EC was further associated with their negative mood (measured by both PANAS-negative and DASS-21) and psychological impact (measured by IES-R) of COVID-19 pandemic. These findings reveal an underlying difference between CC and EC. CC refers to the cognitive ability that enables people to solve problems by using novel and useful ideas (e.g., Sternberg, 1999) while EC is a combination of cognitive abilities and dispositional traits associated with originality and appropriateness of emotional experience (e.g., Averill, 1999; Ivcevic et al., 2007).

Moreover, the current study found different mediation and moderation mechanisms in bilinguals' CC and EC. On the one hand, we found that cognitive flexibility was a simple mediator in the association between L2 proficiency and CC. Adaptive ER strategies did not play any role in this model. On the other hand, the current study also showed that bilinguals' L2 proficiency had a positive influence on their EC through cognitive flexibility only when bilinguals' adaptive ER strategies were playing a moderation role on the indirect influence (i.e., L2 proficiency → cognitive flexibility → EC). We did not find that cognitive flexibility was a simple mediator in the association between L2 proficiency and EC. However, when bilinguals' adaptive ER strategies were considered, we found a moderated mediation mechanism in their EC, suggesting that their L2 proficiency might influence their EC through cognitive flexibility only when the moderation influence of adaptive ER strategies was considered. Therefore, adaptive ER strategies were playing an important moderating role in bilinguals' EC but not CC. The differences between CC and EC we found might be due to the different nature of the two psychological variables (e.g., Zenasni and Lubart, 2008; Martsksvishvili et al., 2017).

### Limitations

There are several limitations as well as suggestions for future research in the current study. First, only young and healthy bilinguals, most of whom were females, participated the current investigation. Future studies should include a balanced participant distribution of the two genders and extend to more diverse participant groups to validate the results of the current study. Second, due to the outbreak of the COVID-19 pandemic, the current study used only online questionnaire surveys. Although the internal consistency reliabilities of those questionnaires were satisfactory, the results we found need to be tested in future experimental research with systematic variable control after the pandemic. Third, the current study tested only Chinese-English bilinguals. The interpretation and the extension of the findings need to proceed with caution. Future studies are needed to test whether bilinguals have a bilingual advantage in both CC and EC over monolinguals using comparative studies.

## Conclusion

The current study found similarities between CC and EC. They both are associated with L2 proficiency, cognitive flexibility, and adaptive ER strategies. The current study further showed different cognitive mechanisms behind the influence of L2 proficiency on CC and EC in bilinguals, and that adaptive ER strategies played a key role in EC. Simple mediation analysis showed that L2 proficiency influenced participants' CC both directly and indirectly through cognitive flexibility. Moderated mediation analysis suggested that L2 proficiency influenced bilinguals' EC both directly and indirectly through cognitive flexibility only when participants' adaptive ER strategies were moderating the indirect influence from L2 proficiency to EC. The findings in the current study imply that adaptive ER strategies would play a crucial role in bilinguals' EC, especially during the time of crisis (e.g., the COVID-19 pandemic).

## Data Availability Statement

At the current stage, the original data are not made openly available in online repositories because of some restrictions in authors' institutions and/or confidentiality requirements of the programs where the authors work. Some of authors' institutional ethics committees require that the data be made available from them for researchers who meet the criteria for access to confidential data. Requests to access the datasets should be directed to Yadan Li at liyadan@snnu.edu.cn.

## Ethics Statement

The studies involving human participants were reviewed and approved by the Academic Committee of the Ministry of Education Key Laboratory of Modern Teaching Technology. The patients/participants provided their written informed consent to participate in this study.

## Author Contributions

YY designed the study, performed the data analysis, and drafted the manuscript. SW reviewed, revised, and proofread the manuscript. JD critically reviewed the content and revised and proofread the manuscript. KJ organized participants' recruitment and collected and analyzed data. YL collected data, reviewed, and revised the manuscript. All authors contributed to the article and approved the submitted version.

## Conflict of Interest

The authors declare that the research was conducted in the absence of any commercial or financial relationships that could be construed as a potential conflict of interest.

## Publisher's Note

All claims expressed in this article are solely those of the authors and do not necessarily represent those of their affiliated organizations, or those of the publisher, the editors and the reviewers. Any product that may be evaluated in this article, or claim that may be made by its manufacturer, is not guaranteed or endorsed by the publisher.
